# Characterizing the HIV/AIDS Epidemic in the United States and China

**DOI:** 10.3390/ijerph13010030

**Published:** 2015-12-22

**Authors:** Ming-Bo Huang, Li Ye, Bing-Yu Liang, Chuan-Yi Ning, William W. Roth, Jun-Jun Jiang, Jie-Gang Huang, Bo Zhou, Ning Zang, Michael D. Powell, Hao Liang, Vincent C. Bond

**Affiliations:** 1 Department of Microbiology, Biochemistry and Immunology, Morehouse School of Medicine, Atlanta, GA 30310, USA; mhuang@msm.edu (M.-B.H.); wroth@msm.edu (W.W.R.); mpowell@msm.edu (M.D.P.); 2 Guangxi Key Laboratory of AIDS Prevention and Treatment, School of Public Health, Guangxi Medical University, Nanning, Guangxi 530021, China; yeligx@163.com (L.Y.); liangbingyu@163.com (B.-Y.L.); ningchuanyi@126.com (C.-Y.N.); johnjeang@foxmail.com (J.-J.J.); katooo@163.com (J.-G.H.); 3 Guangxi Medical Research Center, Guangxi Medical University, Nanning, Guangxi 530021, China; gxzhoubo520@126.com (B.Z.); zangninggxnn@163.com (N.Z.)

**Keywords:** HIV/AIDS epidemic, the United States, China

## Abstract

The HIV/AIDS data from the national surveillance systems of China and the United States from 1985 to 2014 were compared to characterize the HIV/AIDS epidemic in both countries. The current estimated national HIV prevalence rate in China and the United States are 0.0598% and 0.348%, respectively. In the United States, the annual number of new HIV infections has remained relatively stable (~50,000 each year) and has shown a downward trend in recent years. The Chinese national HIV prevalence is still low, and new HIV infections have been contained at a low level (50,000–100,000 each year). However, the epidemic has showed an increasing trend since 2012. By risk group, in both countries, men who have sex with men (MSM), heterosexual sex, and injection drug use (IDU) are the most common modes of transmission of new HIV infections. However, in the United States, MSM is the dominant transmission route, accounting for >60% of new infections; whereas in China, heterosexual sex has now become the dominant route, also accounting for >60% of new infections. A rapid increase in the proportion of HIV cases that were attributed to MSM and an obvious decrease in the proportion of HIV cases attributed to IDU in China in recent years imply that the China’s epidemic is still evolving, to some extent, copying what was experienced in the United States. By age group, the proportions of HIV cases that were attributed to the age group 25–59 were comparable between the two countries. However, the United States had a higher proportion of cases that were attributed to age groups 15–19 and 20–24 than China, indicating that youth account for more infections in the United States. One other fact worth noting: in China there is a significant increase in the number of HIV new infections in individuals over 50 years of age, which results in much higher proportion of cases that were attributed to age groups 60–64 and over 65 in China than those in the United States. By race/ethnicity, in the United States, Blacks/African Americans continue to experience the most severe HIV burden, followed by Hispanics/Latinos. In China, no official data on race/ethnicity disparities are currently available. Thus, region, risk group, age are important factors in the HIV epidemics in both countries.

## 1. Introduction

Human Immunodeficiency Virus (HIV), which causes acquired immunodeficiency syndrome (AIDS), has become one of the world’s most serious health and developmental challenges. The first cases in the United States were reported in 1981. Globally there are now approximately 36.9 million people currently living with HIV and tens of millions of people have died of AIDS-related causes since the beginning of the epidemic [1]. In the United States, since the first cases appeared, new HIV infections (incidence) reached its highest level in the 1980s. This was followed by declines, mostly due to medical advances and programs aimed at prevention and care that reached HIV/AIDS patients or people at risk for HIV. Subsequently, new infections have remained at about 50,000 for more than a decade. Although the response to the epidemic in the United States has yielded numerous successes, challenges remain. The HIV transmission patterns and distribution show that racial and ethnic minorities, different age groups and high-risk populations have been disproportionately affected by HIV/AIDS.

In China, the first AIDS case (an imported case) was found in 1985 in a foreign tourist. The first indigenous cases were identified as an outbreak among heroin abusers in 1989 in Yunnan Province, located in China’s southwest region bordering the southeastern Asian countries Myanmar, Laos, and Vietnam. From then to the mid-1990s, HIV spread gradually to other Chinese regions from Yunnan Province along the drug trafficking routes, also spreading from injection drug users (IDUs) to their sexual partners and children. In the mid-1990s, a second major outbreak was found among commercial plasma donors in the east-central provinces. Simultaneously, HIV was spreading through sexual transmission. By 1998, the HIV/AIDS epidemic had reached all 31 provinces and was in a period of rapid increase. By 2005, the number of estimated HIV infections had reached 650,000 with the numbers increasing annually between 2005 and 2013. However, a reduced growth was observed, due to a number of substantial intervention initiatives that had been introduced since the end of 2003. This included “Four Free and One Care” policy (free antiretroviral drugs, free prevention of mother-to-child transmission, free voluntary counseling and testing, free schooling for children orphaned by AIDS, and care to people living with HIV/AIDS), methadone maintenance treatment (MMT) programmes, and needle exchange programmes (NEPs), *etc*. Overall, China lags about a decade behind the United States in the HIV/AIDS epidemic. The number of annual new infections in the United States has been stable for more than a decade; however, in China it still appears to be on an upward trend. To some extent, the HIV/AIDS epidemic in China is going through what had been a trend experienced previously in the United States. A comparison of the HIV/AIDS epidemic in the two countries will be of constructive significance to help control the epidemic in both countries. More importantly, guidance for China’s epidemic control efforts may be gained from the United States’ experience. Thus, we review and compare the epidemic characteristics of HIV/AIDS in the United States and China using data from the national surveillance systems of China and the United States from 1985 to 2014.

## 2. Methods

We collected China’s HIV/AIDS epidemic data from public data of the National HIV/AIDS Case Reporting System (CRS) and National HIV Sentinel Surveillance System of the Chinese Center for Disease Control (China CDC). The Case Reporting System was established in 1985 to track people living with HIV/AIDS (PLHIV), record case-finding, and inform HIV/AIDS control and prevention strategies. The National HIV Sentinel Surveillance System was established in 1995 to monitor the HIV epidemic trend among most-at-risk populations over time by surveying risk behaviors and testing HIV antibodies. Eight subpopulations were covered in Sentinel Surveillance System: drug users, female sex workers (FSWs), men who have sex with men (MSM), male sexually transmitted disease (STD) clinic attendees, long-distance truck drivers, antenatal care clinic attendees (ANCs), young college students, and migrant workers. Both provincial HIV/AIDS epidemic and demographic data were also collected from the annual working report on China’s AIDS epidemic estimate in 2009, 2011 by the Ministry of Health of China, the Joint United Nations Programme on HIV/AIDS (UNAIDS), World Health Organization (WHO), HIV/AIDS Surveillance Report by The National Health and Family Planning Commission of China, reports at provincial level by local CDCs. HIV/AIDS epidemic data in the United States were collected from published data of the U.S. CDC, National Center for HIV/AIDS, Viral Hepatitis, STD, and TB Prevention, HIV Surveillance Report and HIV basic data by the U.S. CDC.

## 3. Results and Discussion

### 3.1. HIV/AIDS Epidemic in the United States and China

In the United States, the population on 4 July 2013 was 316,148,990. It was estimated that more than 1.1 million people (0.348%) were living with HIV infection, and 15.8% of them were unaware of their infection [2,3,4]. Since the first cases of HIV/AIDS were reported in 1981, over 1.8 million people in the United States are estimated to have been infected with HIV, including over 650,000 who died during that period. While the number of new HIV infections is down from its peak in the 1980s, new infections have remained at approximately 50,000 per year in recent years (Table 1).

**Table 1:** Estimated numbers for HIV/AIDS, by year of diagnosis, United States (2008–2013).

Classification	2008	2009	2010	2011	2012	2013
New HIV infections	51,477	47,897	46,021	44,883	46,154	48,145
AIDS	33,419	31,915	29,168	27,283	26,444	27,135

Adapted from [2,3,4].

In China, the population on January 2013 was 1,354,040,000. By the end of 2013, it was estimated that 810,000 Chinese PLHIV (0.0598%), of whom 173,825 were diagnosed with AIDS (Table 2) [5,6,7,8,9,10,11]. Beginning with the first identified case of HIV in 1985 through the end of 2013, about 937,000 people in China were estimated to be infected with HIV, including over 127,000 reported deaths from AIDS. Compared with the estimate of 810,000 PLHIV in China in 2013, only 436,817 PLHIV cases were reported. The difference between these numbers suggests that a large number of PLHIV have not yet been identified, and are unaware of their infection status, presenting an increased risk for further transmission.

**Table 2:** Estimated numbers for HIV/AIDS in China (2005–2014).

Classification	2005	2007	2009	2011	2012	2013	2014
PLHIV (Estimated)	650,000	700,000	740,000	780,000	- *	810,000	-
PLHIV (Reported)	-	-	272,000	352,000	385,817	436,817	500,679
AIDS	75,000	85,000	105,000	154,000	145,463	173.825	204,683
New HIV infections	70,000	50,000	48,000	48,000	82,434	90,119	103,501
HIV prevalence (Estimated)	0.050%	0.054%	0.057%	0.058%	-	0.06%	-

- *: Not Available; Adapted from [5,6,7,8,9,10,11].

Compared to the stable PLHIV number (1,100,000–1,200,000) in the United States in a recent decade, China’s PLHIV number increased annually in recent years, from 650,000 in 2005 to 810,000 in 2013 (Table 2) [5,6,7,8,9,10,11]. Correspondingly, the reported PLHIV cases and the number of AIDS patients also increased annually, reaching 500,679 and 204,683 in 2014, respectively (Table 2) [5,6,7,8,9,10,11]. The annual new infection number (~50,000) in the United States was also stable from 2008 to 2013. New HIV infections in China remained at approximately 50,000 each year between 2007–2011, but increased to 80,000–100,000 each year in 2012–2014 (Table 2). 

### 3.2. HIV/AIDS Distribution in the United States and China

In the United States, the HIV/AIDS epidemic is not evenly distributed across states and regions. The rate (the number of cases per 100,000 people) of persons living with an AIDS diagnosis was highest in the Northeast, followed by the South, the West, and the Midwest (in 2008, Figure 1) [12]. Regionally, the South accounted for about half of HIV diagnoses. In the United States, HIV/AIDS is concentrated in urban areas. New HIV diagnoses are concentrated primarily in large metropolitan areas, with New York, Los Angeles, and Miami topping the list [12].

**Figure 1:**
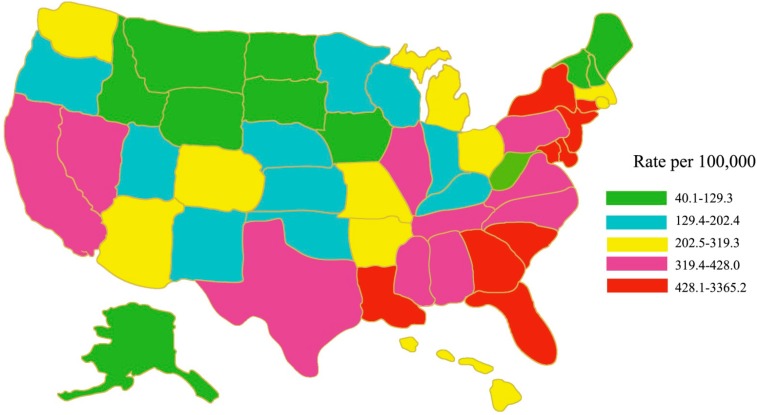
Rates of persons aged 18–64 years living with a diagnosis of HIV infection, year-end 2008—United States. Adapted from [12].

**Figure 2:**
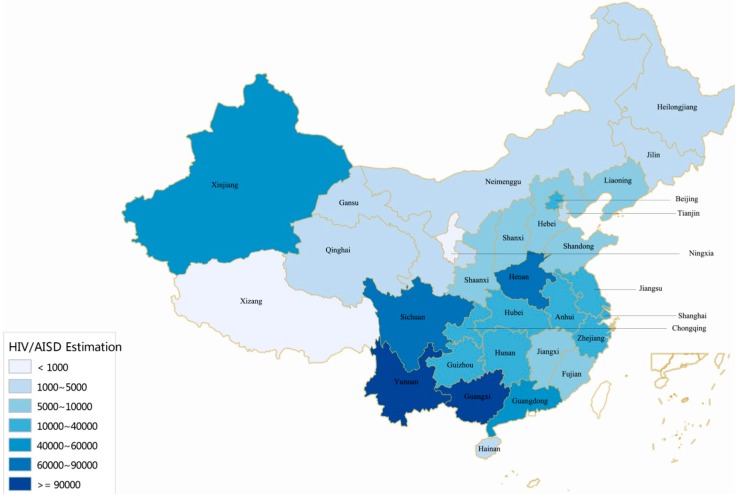
Geographic distribution of the reported 500,679 PLHIV in China (Cumulative number of reported HIV/AIDS cases in different provinces, by October 2014). Adapted from [5,6,7,8,9,10].

In China, the epidemic is also unevenly distributed across provinces and regions (Figure 2). The epidemic is severe in some geographical regions. The twelve provinces with the highest cumulative number of reported HIV/AIDS cases (Yunnan, Guangxi, Sichuan, Henan, Guangdong, Xinjiang, Chongqing, Guizhou, Hunan, Beijing, Zhejiang, and Jiangsu) represent 84.3% of the national total (Figure 2), while the seven provinces with the lowest cumulative number of reported cases (Tibet, Qinghai, Ningxia, Inner Mongolia, Gansu, Hainan, and Tianjin) only account for ~1% of the national total (Figure 2). Southwest and northwest China are the most HIV-affected regions, including provinces of Yunnan, Guangxi, Sichuan, Guizhou, Guangdong, and Xinjiang (Figure 2).

### 3.3. HIV Transmission Patterns

In the United States, MSM, heterosexual, and injection drug use (IDU) were the top three factors for new HIV infections. They accounted for 58.32%, 28.62%, and 8.92% of all new infections in 2009, and 64.81%, 25.17%, and 6.54% in 2013, respectively, with an increasing trend in MSM and a decreasing trend in heterosexuals or IDUs (Table 3) [2,3,4]. MSM including gay, bisexual, and other represent only ~2% of the United States population, yet have been the population most severely affected by HIV. At the end of 2011, an estimated 500,022 (57%) persons living with an HIV diagnosis in the United States were gay and bisexual men, or gay and bisexual men who also inject drugs [2,3,4]. There are few population-based studies of sex workers in the United States because sex work is a stigmatized occupation and is illegal throughout most of the United States and the world. This lack of data and understanding around sex work creates a significant barrier to HIV prevention efforts and other services.

In China, the HIV transmission pattern is different from that in the United States. From 1985 to 2006, HIV transmission has been particularly high among IDUs and former plasma donors (Table 3) [5,6,7]. However, in recent years, heterosexual sex has become the dominant route of transmission. The portion of identified PLHIV that acquired HIV through heterosexual sexual contact has been greatly increasing, from 30.6% in 2006 to 66.4% in 2014 [5,6,7,8,9,10,11]. Consistent with the current situation in the United States, a rapid increase in the proportion of HIV cases that were attributed to MSM (Table 3) was observed in recent years, from 2.5% in 2006 to 25.8% in 2014, representing a 10-fold increase (Table 3) [5,6,7,8,9,10,11]. The reason for a significant increase in this population could be the increased openness of male homosexuality in China. Similar to the decreased trend in the United States, but more obvious and rapid, a downward trend in HIV proportion of HIV cases attributed to IDU has been observed in China since 2005 (Table 3). In China, HIV prevalence also varies greatly among different sub-populations. The HIV incidence among drug users (particularly IDUs) is the highest, and shows clear regional disparities. The places with the highest percentage of HIV cases among drug users are located in the southwest and northwest China. The HIV proportion among FSWs has remained relatively low nationally [5,6,7,8,9,10,11]. HIV prevalence exceeded 1% in FSWs sentinel surveillance sites located in the Yunnan, Xinjiang, Guangxi, Sichuan and Guizhou provinces/autonomous regions [5,6,7]. However, a higher prevalence (e.g., some around or over 1%) has been reported in several sentinel surveillance sites in areas most impacted by the epidemic [5,6,7].

**Table 3:** Distribution of transmission routes of reported HIV/AIDS cases in the United States and China (1985–2014).

Year	Annual Transmission Constitute of Reported (%)						
	Country	Heterosexual	Homosexual	IDU *	Homosexual and IDU **	MTCT ***	Blood and Others
1985–2005	USA	- ****	-	-	-	-	-
	China	11.30	0.30	44.20	-	1.10	43.10
2006	USA	-	-	-	-	-	-
	China	30.60	2.50	34.10	-	1.50	31.30
2007	USA	-	-	-	-	-	-
	China	38.90	3.40	29.20	-	1.50	27.00
2008	USA	-	-	-	-	-	-
	China	40.30	5.90	27.90	-	1.30	24.60
2009	USA	28.62	58.32	8.92	3.43	0.38	0.33
	China	47.10	8.60	25.80	-	1.40	17.10
2010	USA	27.82	60.06	8.04	3.34	0.40	0.34
	China	54.90	10.80	22.10	-	1.30	10.90
2011	USA	26.94	62.12	7.19	3.06	0.32	0.37
	China	62.60	16.10	15.60	1.10	1.30	3.30
2012	USA	25.59	63.96	6.68	2.91	0.36	0.50
	China	68.00	19.10	9.30	0.70	1.00	1.90
2013	USA	25.17	64.81	6.54	2.68	0.23	0.57
	China	69.40	21.40	7.20	0.50	0.90	0.55
2014	USA	-	-	-	-	-	-
	China	66.40	25.80	5.60	0.40	0.70	1.08

IDU *: injection drug use; Homoexual and IDU **: individuals reporting homosexual activity and injection drug use; MTCT ***: mother-to-child transmission; - ****: not available; Adapted from [5,6,7,8,9,10,11,12].

### 3.4. Risk by Age Group

**Figure 3:**
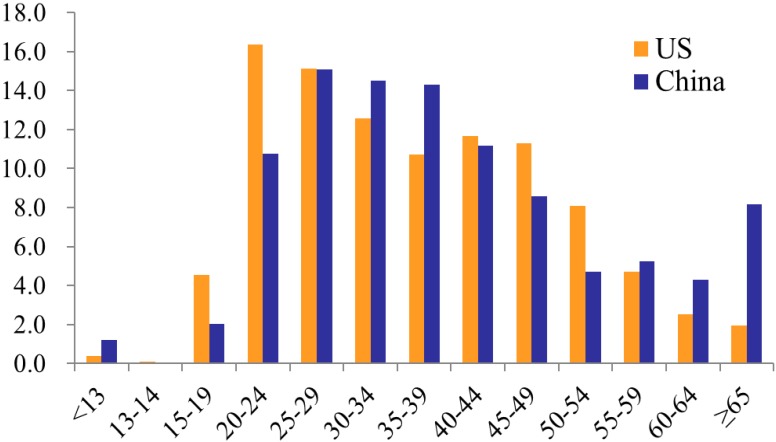
Comparison of distribution of HIV cases by age, 2011, United States and China. Source: [3,5].

The distribution of HIV cases by age was somewhat different between the United States and China according to the data in 2011 (Figure 3) [3,5]. The proportions of HIV cases that were attributed to persons aged 25–59 were comparable between the two countries. However, a higher proportion of HIV cases attributed to the 15–19 and 20–24 year age groups in the Unite States, relative to China; while in China there was a higher proportion of cases in the 55 and older age group relative to the United States (Figure 3). In the United States, youth account for a substantial number of HIV infections. In 2011, youth aged 13 to 24 made up 17% of the United States population, but accounted for an estimated 21.01% of all new HIV infections; and this data increased to 25.5% in 2013 [13]. Americans aged 50 and older have many of the same HIV risk factors as younger Americans. A growing number of people aged 50 and older in the United States are living with HIV infection, accounting for 26%, of the estimated 1,100,000 people living with HIV infection in the United States in 2011 [12,13]. In China, there was an unexpected increase in the number of reported cases in individuals aged 50 and older in recent years. The proportion of the total annual number of reported cases attributed to the 50–64 age groups increased from 1.6% in 2000 to 14.3% in 2011, representing a 9-fold increase [5,6,7]. The proportion of the total annual number of reported cases made up by the age group 65 years old and above increased from 0.34% to 8.16%, representing a 24-fold increase during the same period [5,6,7].

### 3.5. Race/Ethnicity

**Figure 4:**
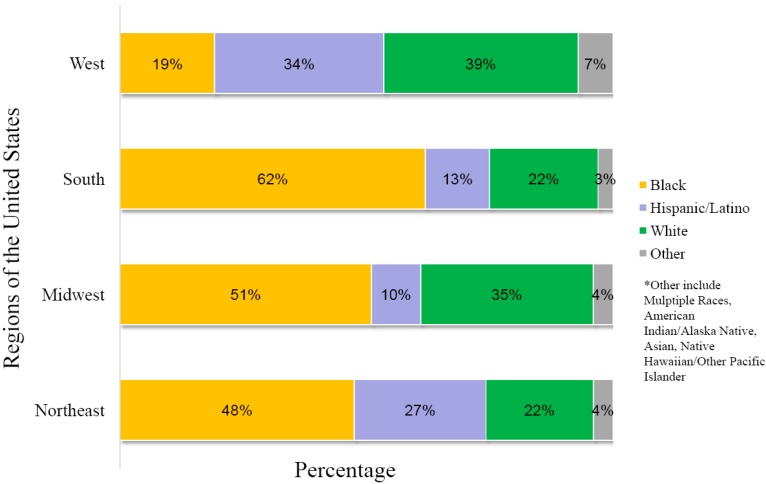
AIDS in the United States, Race/Ethnicity of Persons Diagnosed with AIDS in 2010 in the 50 States and District of Columbia, by Region of Residence. Adapted from [12].

In the United States, Blacks/African Americans are the racial/ethnic group most affected by HIV. African Americans accounted for an estimated 44% of new HIV infections in 2010 [13]. The rate of new HIV infection in African Americans is eight times that of whites based on population size. Gay and bisexual men accounted for most new infections among African Americans in 2010 [13]; young gay and bisexual men aged 13 to 24 are the most affected of this group. Hispanics/Latinos are also disproportionately affected by HIV, accounting for 21% of new HIV infections in 2010. The approximately 5.2 million American Indians and Alaska Natives, who represent about 1.7% of the US population, ranked fifth in estimated rates of HIV infection diagnoses, with lower rates than in Blacks/African Americans, Hispanics/Latinos, Native Hawaiians/Pacific Islanders, and people reporting multiple races, but with higher rates than in Asians and whites [13]. The number of HIV diagnoses among Asians has increased in recent years, along with the increased growth of the Asian population in the United States. An analysis of AIDS cases among racial/ethnic groups (Figure 4) indicates that the region of the United States with the highest percentage of African Americans diagnosed with AIDS in 2010 was the Southeast, while the Western US had the greatest percentage of Hispanic/Latin Americans diagnosed with AIDS. The greatest proportion of non-Hispanic whites diagnosed with AIDS were in the Northeast and Midwest US. These percentages are likely related to differences of the racial/ethnic populations in the various regions of the country. In China, there are currently no official HIV infection data correlated with race/ethnicity. Nevertheless, the fact that four (Yunnan, Guangxi, Sichuan, Xinjiang) of the six provinces with the highest cumulative number of reported HIV/AIDS cases in China (representing 75.8% of the national total) are also areas where there are large populations of ethnic minorities suggests that HIV infection as it correlates with ethnicity should be a focus in future HIV surveillance in China.

## 4. Conclusions

Although China’s current estimated national HIV prevalence rate (0.0598%) is lower than in that of the United States (0.348%), compared to the relatively stable HIV/AIDS epidemic in the United States, China’s HIV/AIDS epidemic is diverse and evolving rapidly. In both countries, MSM, heterosexuals, and IDU are the most common modes of transmission of new HIV infections, with MSM being the dominant route of transmission (>60%) in the United States and heterosexual transmission being the dominant route (>60%) in China. A rapid increase in the proportion of HIV cases attributed to MSM in China in recent years suggests that China faces similar challenges to those in the United States. The HIV epidemic has had an effect on different age groups in both countries. In the United States, youth account for a substantial number of HIV infections, and a growing number of people aged 50 and older is found to be infected with HIV. Larger increases were observed in China *versus* the United States in the number of new HIV infections in individuals over 50 years old. Finally, race/ethnicity plays a more prominent role in the HIV epidemic in the United States compared with China. Since there is a paucity of data on ethnicity and HIV infection in China, the information presented in this report is likely to change as more data becomes available. Thus, analysis of the HIV epidemic in China is seen as an area for future study.
